# Dietary Aroclor 1254-Induced Toxicity on Antioxidant Capacity, Immunity and Energy Metabolism in Chinese Mitten Crab *Eriocheir sinensis*: Amelioration by Vitamin A

**DOI:** 10.3389/fphys.2019.00722

**Published:** 2019-06-12

**Authors:** Dexiang Feng, Xiaodan Wang, Erchao Li, Xianyong Bu, Fang Qiao, Jianguang Qin, Liqiao Chen

**Affiliations:** ^1^Laboratory of Aquaculture Nutrition and Environmental Health, School of Life Sciences, East China Normal University, Shanghai, China; ^2^Department of Aquaculture, College of Marine Sciences, Hainan University, Haikou, China; ^3^College of Science and Engineering, Flinders University, Adelaide, SA, Australia

**Keywords:** polychlorinated biphenyl (PCB), Vitamin A, *Eriocheir sinensis*, oxidative stress, immunity, detoxification enzyme, energy metabolism, mRNA expression

## Abstract

Effects of dietary Polychlorinated biphenyl (PCB) exposure and dietary vitamin A supplementation on Chinese mitten crab *Eriocheir sinensis* were studied with the aim to explain dietary PCB toxicity and toxic alleviation by vitamin A intake in crab. Four diets were used including three experimental diets containing 0, 80000 or 240000 IU/kg vitamin A with each experimental diet containing 10 mg PCB/kg diet, and a control diet (without vitamin A and PCB supplementation) in 56 days feeding trial. Crabs fed the PCB-only diet had significantly lower weight gain than those fed the control diet. No significant difference was observed in crab survival among all groups. Crabs fed the PCB-only diet had a significantly higher malondialdehyde content and antioxidase superoxide dismutase activity in the serum and hepatopancreas, and higher erythromycin N-demethylase and glutathione S-transferase activities in the hepatopancreas than those fed the control diet. However, supplementation of dietary vitamin A decreased the levels of all these parameters. The hepatopancreatic cytochrome P450 2 and 4 (CYP2, CYP4), fatty acid binding proteins 3 and 10 (FABP3, FABP10) and intracellular lipolytic enzyme (IL) Messenger Ribonucleic Acid (mRNA) levels in the PCB-only group were significantly higher than those in the control group, and dietary 240000 IU/kg vitamin A supplementation decreased hepatopancreatic CYP4, FABP3, FABP10 and IL enzyme mRNA level. The crabs fed 80000 IU/kg vitamin A supplementation diet had the highest level of retinoid X receptor mRNA in the hepatopancreas. The structure of the hepatopancreas was damaged and the deposit of lipid droplets decreased with dietary PCB exposure. Both levels of vitamin A supplementation alleviated the damage and increased lipid droplets in the hepatopancreas. Dietary PCB exposure significantly reduced total hemocyte count (THC), and phenoloxidase, acid phosphatase activities in the serum. Post-challenge survival of crab in the experimental PCB-only diet group was low compared with that in the control. Supplementation of 240000 IU/kg vitamin A significantly increased the THC and phenoloxidase activity in the serum and post-challenge survival compared with those in the PCB-only group. This study indicates that dietary vitamin A can improve the antioxidant capacity, immune response, detoxification enzymes activities, energy metabolism and hepatopancreas tissue structure of Chinese mitten crab fed PCB contaminated diets.

## Introduction

Polychlorinated biphenyls (PCBs) are a ubiquitous persistent organic pollutant (POP) and is harmful to organisms, while Aroclor 1254 is a commercial PCB manufactured to obtain a specific level of chlorination as a mixture of many PCB congeners. Although the use of PCB has been banned since 1970s, PCBs are still present in the environment in most industrialized countries because they can be transferred, bioaccumulated and biomagnified in the food web (Ulbrich and Stahlmann, 2004). PCBs can cause various adverse effects on many species. With regards to aquatic animals, toxic effects of PCB have been reported in Atlantic killifish (*Fundulus heteroclitus*, Glazer et al., 2018), Rainbow trout (*Oncorhynchus mykiss*, Fritsch and Pessah, 2013) and Sea bass (*Dicentrarchus labrax*, Schnitzler et al., 2011). Farmed crustaceans are easily at risk of PCB both in the environment and their food organisms (Han et al., 2018). However, there is a lack of information on the effects of dietary PCB on crustaceans, which may cause a loss of millions of dollars in aquaculture industry.

Vitamin A is involved in several physiological functions such as growth, development, reproduction and immunity, and prevent lipid peroxidation in the body due to its active chemical properties, participate in the endocrine cycle in organisms (Debier and Larondelle, 2005). In addition, vitamin A and PCB have the same metabolic pathway and both are metabolized by cytochrome P450, and thus PCB and vitamin A could interact with each other in an organism (Vanden Berghe et al., 2013; Berntssen et al., 2016). Addition of retinol dehydrogenase inhibitors can improve heart developmental malformation induced by PCB in Zebrafish (Li et al., 2014). Furthermore, dietary vitamin A supplementation can regulate the level of CYP, UDPGT, GST gene expression and enzyme activities caused by PCB exposure (Arukwe and Nordbø, 2008). Similarly, studies have shown that vitamin A can alleviate oxidative stress and energy metabolism abnormalities caused by PCB exposure and protect damage from Aroclor 1254-induced toxicity in the hepatocytes of Chicken embryos (Zhou and Zhang, 2005).

The Chinese mitten crab *Eriocheir sinensis* is an important species in aquaculture and its production reached over 750 000 tons with a value over 5.5 billion US dollars in China, 2018 (China’s Ministry of Agriculture, 2018). However, with the increasing human activities, *E. sinensis* are at risk of PCB in the environment and through their food components such as fish oil, broken corn and commercial feed (Han et al., 2018). Thus, PCB has become a serious threat to crab health and survival in aquaculture. But there is a lack of information of PCB toxicity on *E. sinensis*. The optimum dietary vitamin A requirement of juvenile Chinese mitten crab is between 69740–70770 IU/kg (Sun et al., 2009). Based on previous research results, we hypothesized that dietary vitamin A supplementation may alleviate the toxic effects of PCBs in Chinese mitten crab. Therefore, in present research, we aimed at investigating the effect of dietary PCB (aroclor1254)-induced toxicity on growth, antioxidant capacity, immunity and energy metabolism, and alleviative the toxic effects by adding vitamin A in the diet through nutrition regulation.

## Materials and Methods

### Experimental Diets

The polychlorinated biphenyl as Aroclor 1254 (CAS No. 11097-69-1) was purchased from AccuStandard Co. (purity > 99%, New Haven, United States). Four isonitrogenous and isoenergetic experimental diets were formulated. The ingredients and nutrient composition of the experimental diets are shown in Table 1. The four experimental diets contained 0 (PCB-only), 80000 (PVA80K) or 240000 (PVA240K) IU/kg vitamin A with each experimental diet containing 10 mg/kg Aroclor1254 diet, and a control diet (without vitamin A and PCB supplementation). The feed pellets were extruded into 1.5-mm diameter by a double helix plodder (F-26, South China University of Technology Industrial Factory, Guangdong, China). The diet pellets were then dried through a blower at room temperature to contain < 10% moisture. The diets were stored at −20°C until feeding.

**Table 1:** Ingredients and nutrients in the diets (%).

Ingredients	Experimental diets			
	**Control**	**PCB-only**	**PVA80K**	**PVA240K**
Fish meal	23	23	23	23
Casein	20	20	20	20
Fish oil	6	6	6	6
Gelatin	8	8	8	8
Lecithin	0.5	0.5	0.5	0.5
Cholesterol	0.5	0.5	0.5	0.5
Corn starch	25	25	25	25
Vitamin mix	4	4	4	4
Mineral mix	3	3	3	3
CMC-Na	2	2	2	2
Choline chloride	0.5	0.5	0.5	0.5
Cellulose	7.5	7.5	7.5	7.5
Vitamin A supplement	0	0	80000IU	240000IU
Aroclor1254	0mg/kg	10mg/kg	10mg/kg	10mg/kg

*Vitamin mixture:(mg kg^−1^ diet): rerinol acetate, 26; calcium pantothenate, 204; riboflavin, 33; nicotinic acid, 10.7; pyridoxine hydrochloride, 60; α-tocopherol acetate, 20; thiamine hydrochloride, 21; menadione, 59; cyanocobalamin, 0.3; cholecalciferol, 0.15: folic acid,12; biotin, 3.6; inositol, 4000. All ingredients were diluted with α-cellulose to 1 g. Mineral mixture (g kg^−1^ diet):NaH_2_PO_4_, 6.45; Ca(H_2_PO_4_)_2_⋅H_2_O, 7.95; CaCO_3_, 3.15; Ca(CH_3_CHOHHOOC)_2_⋅5H_2_O, 4.95; KH_2_PO_4_, 3; KCL, 0.84; KI, 0.0069; MgSO_4_⋅7H2O, 3; FeC_6_H_5_O⋅nH_2_O, 0.3; CuCl_2_⋅2H_2_O, 0.0189; AlCl_3_⋅6H_2_O, 0.0072; ZnSO_4_⋅7H_2_O, 0.1428; MnSO_4_⋅H_2_O, 0.0321; CoCl_2_⋅6H_2_O, 0.042. Attractant^c^: (% of attractant): Gly,0.6; Ala, 0.6; Glu,0.6; Betaine, 1.2. CMC, Carboxy methyl cellulose sodium.*

### Experimental Crab and Rearing

Juvenile *E. sinensis* were obtained from a local crab company, Shanghai, China. All crabs were acclimated in tanks (300 L) in the Biological Station of East China Normal University for 7 days. Six hundred healthy crabs with mean weight (15.0 ± 1.2 SD) g were randomly assigned to 12 tanks (250 L) with corrugated plastic pipes (12 cm long and 25 mm diameter) as shelters to reduce attacking behavior. There were four treatments with three replicate tanks each. Each replicate tank contained 25 crabs. During the experiment, crabs were fed twice daily between 08:00–08:30 and 18:00–18:30 h, and the daily ration was adjusted to be slightly over satiation based on the amount of feed leftover on the previous day. Two hours after feeding, uneaten feed was removed with a siphon tube. Daily water exchange rate was 1/3 of the tank volume. The incoming fresh water was aerated thoroughly before entering the water recirculation system. The concentration of NO_2_-N was measured and adjusted every 8 h through water exchange rate. The water quality parameters across all feeding treatments were maintained at 22.0–25.6°C, 7.2–8.7 pH, dissolved oxygen > 8.5 mg/L and ammonia *N* < 0.05 mg/L. The trial lasted 56 days.

### Challenge Test

At the end of the feeding trial, 30 crabs from each treatment were randomly assigned to three tanks. According to the preliminary experiment, crabs were challenged with 0.1 ml of suspension with *Aeromonas hydrophila* (10^7^ CFU). After 48 h, the number of surviving individuals was recorded.

### Sample Collection, Measurement and Analysis

At the end of the 56 days feeding experiment, crabs were fasted for 24 h. All crabs were counted and individually weighed (g) by an electronic balance (LQ-A10002, Yaoxin, China). Crabs were anesthetized on ice for 10 min. Hemolymph was sampled with 1-mL syringe on the leg joints of six crabs in each tank. After incubation at 4°C for 24 h, the serum was separated from the hemolymph by centrifugation (5415R, Eppendorf, Germany) at 4500 rpm and 4°C for 10 min and stored at −80°C for enzyme activity analysis. Part of hemolymph samples was used to count total hemocytes. The crabs were dissected to obtain the hepatopancreas, and the part of hepatopancreas was fixed in buffered formalin prior to histological analysis. The other part of the hepatopancreas was stored at −80°C for further biochemical and molecular analysis. The research protocol was approved by the Committee on the Ethics of Animal Experiments of East China Normal University (E20120101).

### Growth Performance

The indexes for the assessment of growth performance were calculated as follows:

$$\mathrm{Weight gain WG}\%=\frac{\mathrm{final weight}-\mathrm{initial weight}}{\mathrm{initial weight}}*100$$

$$\mathrm{Survival}\;(\mathrm{SR}\%)=\frac{\mathrm{final crab number}}{\mathrm{inital crab number}}*100$$

### Total Hemocyte Counts (THC)

Total hemocyte count were obtained on a hemocytometer (XB.K.25, Qiujing, China). Each hemolymph sample was repeated three times and the mean value was recorded for statistical analysis.

**Table 2:** Primer sequences.

Gene name	Q-PCR primers	Sequence(5^′^–3^′^)	Gene bank No.
CYP2	F	GCTCGAGTACCTCATGCCAGCT	KY292413
	R	TGGTGCTCGATCTCCTCAAAGA	
CYP4	F	GCCAGCCGCTTCTTCTTG	KT027984.1
	R	ACCTCCGCATCCTTCACG	
FABP3	F	CCACCGAGGTCAAGTTCAAGC	KJ804230
	R	TCACACCATCACACTCCGACAC	
FABP9	F	GGGCAACAAAATGACCCATAAG	HM459893
	R	TGGCGAACACGCACAATCCT	
FABP10	F	TGATTGGCTCAGTGCTGTGGGT	GU568242
	R	GGTGTTGGTGAAGTTCTTGTCGC	
IL	F	CGATCCTACGAGTTCTTCA	preliminary trials
	R	GCACTTGGTGTTGTCATC	
RXR	F	TGGAGGACGGCATTGTGCT	KF179131.1
	R	TTCATCTTGGCCACCAGCTCA	
β-actin	F	AGCGCAAGTACTCCGTCTGGAT	HM053699.1
	R	AATGGCAGGGCCAGACTCAT	

### Biochemical Analysis

The superoxide dismutase (SOD), catalase (CAT), and glutathione peroxidase (GPx) activities and malondialdehyde (MDA) content of serum and hepatopancreas were measured by using commercial assay kits (Nanjing Jiancheng Bioengineering Institute, China) in accordance with the instructions of the manufacturer.

The activities of ethoxyresorufin-O-deethylas (EROD), erythromycin N-demethylase (ERND), aminopyrine N-demethylase (APND), uridine 5′-diphospho-glucur-onosyltransferase (UDPGT) and glutathione S-transferase (GST) of hepato-pancreases were measured by using commercial ELISA assay kits (Shanghai Hengyuan Biological Technology Co., Ltd., China) in accordance with the instructions of the manufacturer. The acid phosphatase (ACP), and lysozyme (LZM) activities in the serum were also tested by commercial kits (Nanjing Jiancheng Bioengineering Institute, China).

The Phenoloxidase (PO) activity was measured as described by Ashida (1971). Briefly, 10 μL serum, 300 μL phosphate buffer (0.1 M, pH 6.0), and 10 μL dihydroxyphenylalanine solution (0.01 M) were added to 96-well microtiter plates, and the absorbance [optical density at 490 nm (OD_490_)] was determined every 2 min. An increase of 0.001/minute in the OD_490_ was regarded as 1 unit of activity.

### Analysis of Gene Expression in Hepatopancreas

Total RNA was isolated from the hepatopancreas in three replicates with TRIpure Reagent (RN0101, Aidlab, China). The purity of RNA was tested on a Nano Drop 2000 spectrophotometer (Thermo Fisher, United States). Only the RNA samples with the A260/A280 ratio between 1.8 and 2.0 were used for the subsequent analysis. A PrimeScript^TM^ RT master mix reagent kit with a gDNA eraser (Perfect Real Time, Takara, Japan) was used to synthesize cDNA for quantitative real time-PCR (RT-qPCR). The RT-PCR was performed with the CFX96 Real-Time PCR system (Bio-rad, Richmond, CA, United States). The specific primers for the genes were designed with Primer Premier Software 6.0 according to the *E. sinensis* sequences (Table 2). The intracellular lipolytic enzyme (IL) was designed and validated by Primer Premier 6.0 according to the sequence information in our preliminary trials. The reactions were done in a volume of 10 μL containing 0.5 μL of 10 mM each of forward and reverse primers (1 mmol/L), 2.5 μL of diluted cDNA (1:5 diluted) and 5 μL 2 × SYBR Premix Ex TaqTM and 1.5 μL of RNA-free H_2_O. The programmed reaction steps included 94°C for 3 min, followed by 40 cycles at 94°C for 15 s and 58°C for 50 s, and 72°C for 20 s. And melt curve has been adopted to monitor PCR amplification specificity. Data were quantified by the 2^−ΔΔCT^ method and were subjected to statistical analysis.

### Histological Assay

The hepatopancreas was dehydrated in ethanol, cleaned in toluene, equilibrated in xylene, and embed in paraffin to make solid wax blocks. Then, the embedded hepatopancreas was sectioned with a rotary microtome at approximately 5-μm thick and stained by using haematoxylin and eosin (HE) and oil-red O. The tissue slices were observed on an Axioskop microscope (BX51, Olympus, Tokyo, Japan).

### Statistical Analysis

All data were test for normality and homogeneity of variances by Levene’s test. *T*-test was used between the control and the PCB-only group to test if dietary PCB supplementation could have a significant impact on the performance of crabs. If there was a significant difference, the asterisk (^∗^) was displayed (*P* < 0.05). Then, a one-way ANOVA was used between PCB-only, PVA80K, and PVA240K groups to test if supplementation of vitamin A could alleviate the toxic impact of PCB on crabs, using SPSS 23.0 (IBM, Armonk, NY, United States) followed by Duncan’s multiple range tests. Data were presented as mean ± standard error (SE) with different superscript letters to indicate significant differences between groups. A significant level of *P* < 0.05 was employed in all cases.

## Results

### Growth and Survival

**FIGURE 1:**
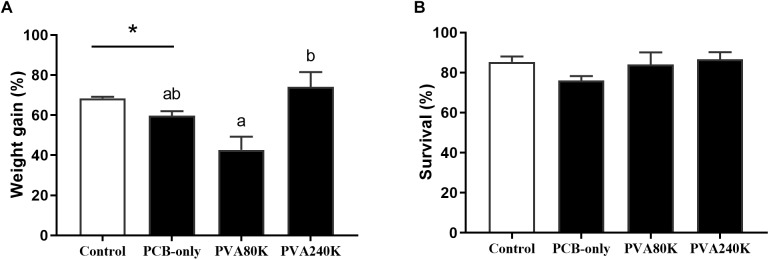
Effects of the vitamin A on the Aroclor 1254-mediated weight gain **(A)** and survival **(B)**; blank bar: diet without PCB from three individuals (*n* = 3); dark bar: diets with PCB. ^∗^*p* < 0.05. Data shown as means ± SEM.

The crabs in PCB-only group had significantly lower weight gain than those in the control group (*P* < 0.05, Figure 1A). Moreover, dietary vitamin A supplementation in the PVA80K and PVA240K groups attained comparable results in weight gain with the PCB-only group (*P* > 0.05). No significant difference was observed in percentage survival of crabs among all groups (*P* > 0.05, Figure 1B).

### Antioxidant Capacity of Serum and Hepatopancreas

**FIGURE 2:**
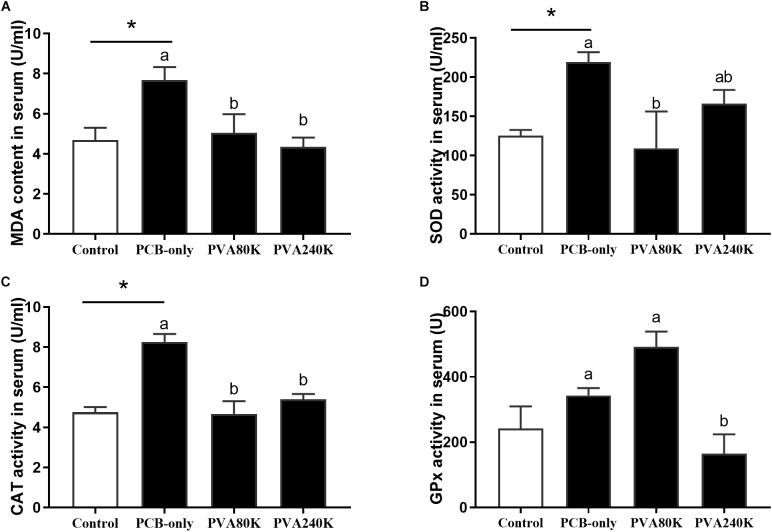
Effects of the vitamin A on the Aroclor 1254-mediated lipid peroxidation (malondialdehyde MDA) **(A)** and activities of superoxide dismutase (SOD) **(B)**, catalase (CAT) **(C)**, and Glutathione peroxidase (GPx) **(D)** of serum from three individuals (*n* = 3); blank bar (diet without PCB), dark bar (diets with PCB). ^∗^*p* < 0.05. Data shown as means ± SEM.

**FIGURE 3:**
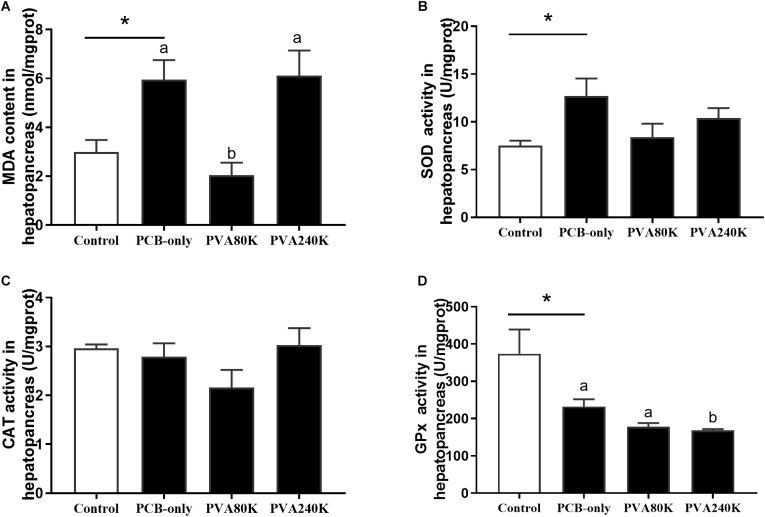
Effects of the vitamin A on the Aroclor 1254-mediated lipid peroxidation (malondialdehyde MDA) **(A)** and activities of superoxide dismutase (SOD) **(B)**, catalase (CAT) **(C)**, and Glutathione peroxidase (GPx) **(D)** of hepatopancreas from three individuals (*n* = 3); blank bar (diet without PCB), dark bar (diets with PCB). ^∗^*p* < 0.05. Data shown as means ± SEM.

SOD activity and MDA content of the serum and hepatopancreas were significantly higher in the PCB-only group than in the control (*P* < 0.05, Figure 2, 3). Crabs fed PVA80K had significantly lower SOD activities and MDA content than those in the PCB-only group (Figure 2A,B, 3A). Crabs fed PCB-only had significantly higher CAT activity in the serum and lower GPx activity in the hepatopancreas than in the control (*P* < 0.05), and both levels of vitamin A supplementation decreased CAT activity in the serum (Figure 2C).

### Activities and Gene Expression of Detoxification Enzymes in Hepatopancreas

**FIGURE 4:**
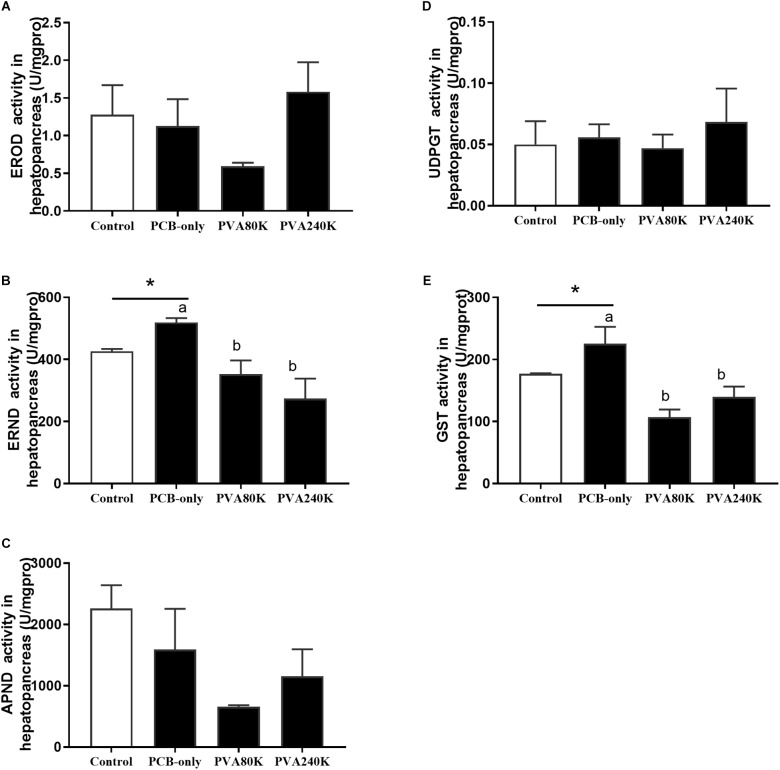
Effects of the vitamin A on the Aroclor 1254-mediated activities of phase I detoxication enzymes: ethoxyresorufin-O-deethylas (EROD) **(A)**, erythromycin N-demethylase (ERND) **(B)**, aminopyrine N-demethylase (APND) **(C)** and phase II detoxication enzymes, uridine 5′-diphospho-glucur-onosyltransferase (UDPGT) **(D)** and glutathione S-transferase (GST) **(E)** of hepatopancreas from three individuals (*n* = 3); blank bar (diet without PCB), dark bar (diets with PCB). ^∗^*p* < 0.05. Data shown as means ± SEM.

No significant differences were observed in the activities of EROD, APND, and UDPGT in the hepatopancreas among all feeding groups (*P* > 0.05, Figure 4), but crabs fed PCB-only had significantly higher ERND and GST activities in the hepatopancreas than in the control (*P* < 0.05, Figure 4B,E). Crabs fed the diet with vitamin A supplementation (PVA80K and PVA240K groups) had significantly lower ERND and GST activities in the hepatopancreas than those fed the PCB-only diet (*P* < 0.05).

**FIGURE 5:**
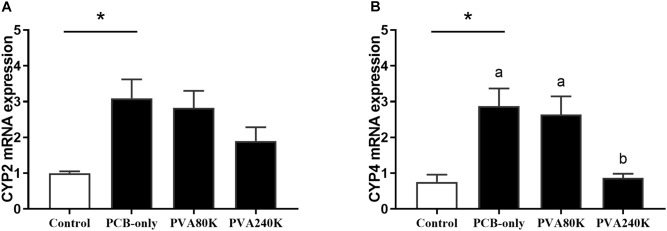
Effects of the vitamin A on the Aroclor 1254-mediated mRNA relative expression of cytochrome P450 2 (CYP2) **(A)** and cytochrome P450 4 (CYP4) **(B)** from three individuals (*n* = 3); blank bar (diet without PCB), dark bar (diets with PCB). ^∗^*p* < 0.05. Data shown as means ± SEM.

The CYP2 and CYP4 mRNA levels of hepatopancreas in the PCB-only group were significantly higher than in the control (*P* < 0.05, Figure 5). Crabs fed PVA240K had significantly lower CYP4 mRNA level than that in the PCB-only group (*P* < 0.05, Figure 5B).

### Gene Expression of Energy Metabolism Related in Hepatopancreas

**FIGURE 6:**
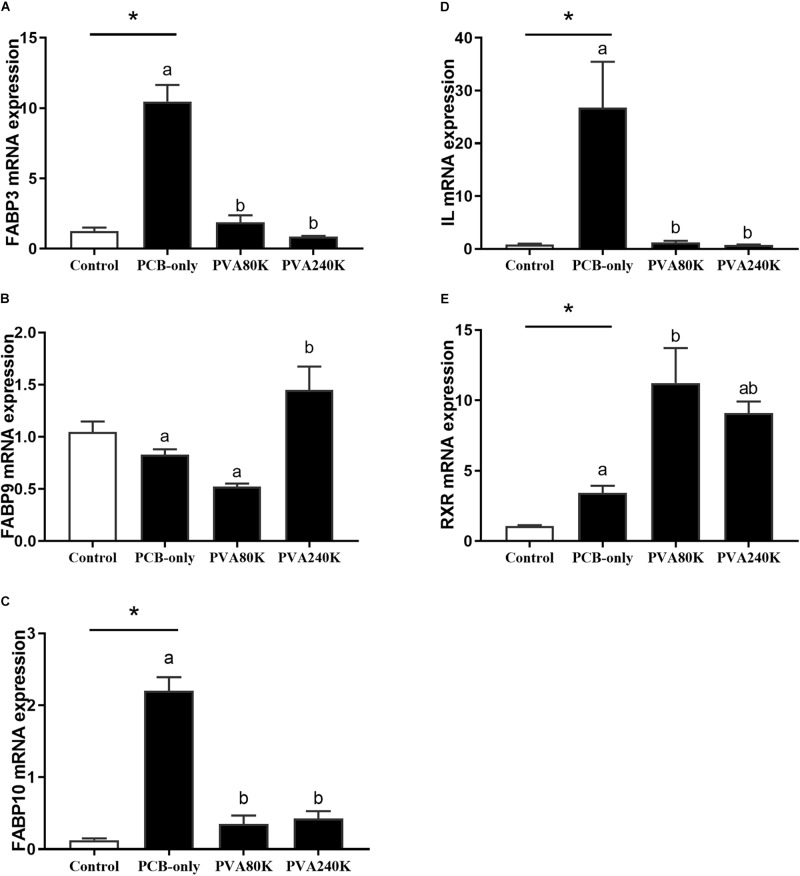
Effects of the vitamin A on the Aroclor 1254-mediated mRNA relative expression of lipid metabolism related genes: fatty acid binding proteins (FABP3) **(A)**, (FABP9) **(B)**, (FABP10) **(C)**, Intracellular lipolytic enzyme (IL) **(D)**, and retinoid X receptor (RXR) from three individuals (*n* = 3) **(E)**; blank bar (diet without PCB), dark bar (diets with PCB). ^∗^*p* < 0.05. Data shown as means ± SEM.

Crabs fed PCB diets had significantly higher FABP3, FABP10, IL and RXR mRNA levels in the hepatopancreas than in the control (*P* < 0.05, Figure 6). With exception of RXR, the expression levels of these genes were significantly decreased when vitamin A was supplemented in the diet compared with those in the PCB-only group (*P* < 0.05). No significant difference was observed in the FABP9 mRNA level in the hepatopancreas between the PCB-only group and the control (*P* > 0.05).

### Histological Assay of Hepatopancreas

**FIGURE 7:**
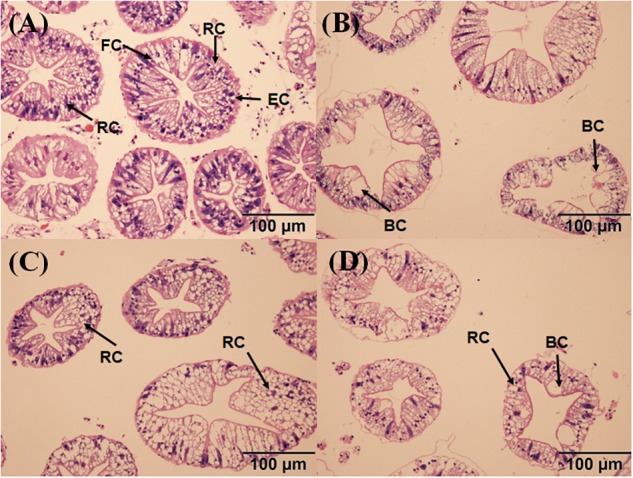
Effects of the vitamin A on the Aroclor 1254-mediated microstructure of hepatopancreas; control group **(A)**, PCB-only group **(B)**, PVA80K group **(C)**, PVA240K group **(D)**; resorptive cell RC (R cell), blister-like cell BC (B cell), fibrillar cell FC (F cell).

**FIGURE 8:**
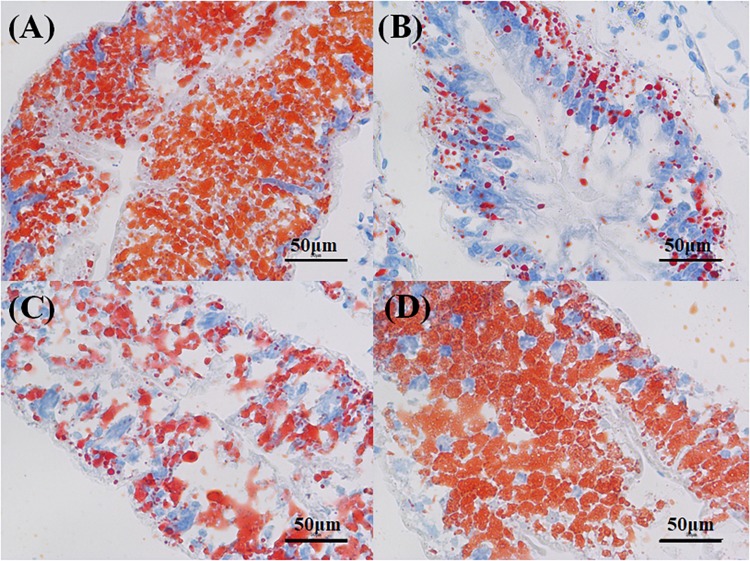
Effects of the vitamin A on the Aroclor 1254-mediated lipid droplets of hepatopancreas; control group **(A)**, PCB-only group **(B)**, PVA80K group **(C)**, PVA240K group **(D)**.

The hepatopancreas of the crabs in the control had a small interstitial space, and the cells were closely arranged in a normal structure (Figure 7A). The massive quantity of R cells was also found in the control group. In the PCB-only group, the damaged cell structure was found, and the lumen space was very large (Figure 7B). In the PVA80K group, more R cells were found compared with those in the PCB-only group (Figure 7C). In addition, a small number of B cells and R cells were observed in the PVA240K group (Figure 7D). The oil-red O staining showed that dietary PCB supplementation decreased the deposit of lipid droplets, and the lipid droplets were increased by vitamin A supplementation in the diet (Figure 8).

### Immune Status and Disease Resistance

**FIGURE 9:**
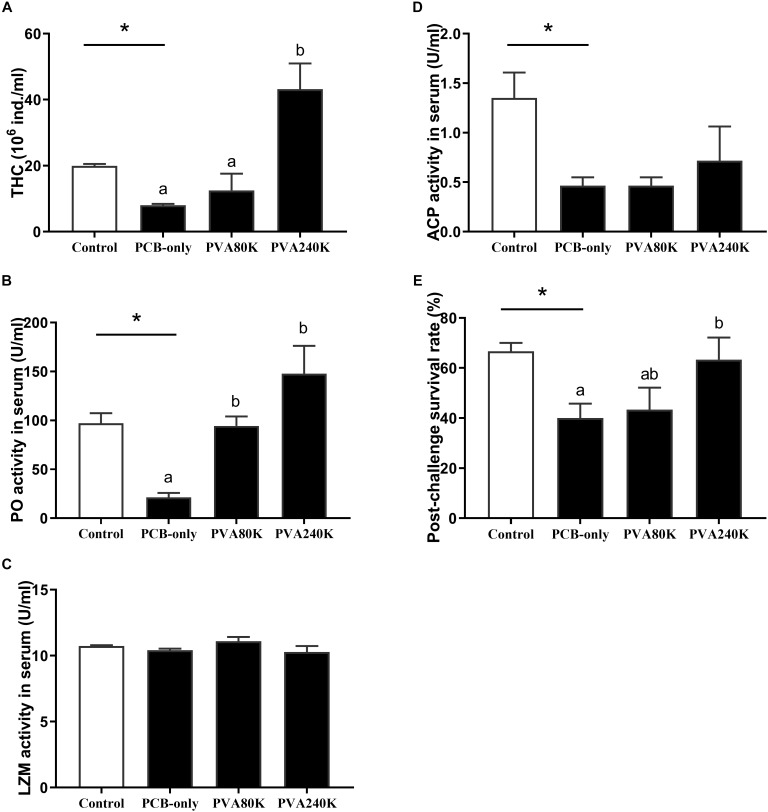
Effects of the vitamin A on the Aroclor 1254-mediated total hemocyte counts (THC) **(A)**, Phenoloxidase (PO) **(B)**, lysozyme (LZM) **(C)** and acid phosphatase (ACP) **(D)** activities in the serum and Post-challenge survival rate **(E)** from three individuals (*n* = 3); blank bar: diet without PCB, dark bar: diets with PCB. ^∗^*p* < 0.05. Data shown as means ± SEM.

Crabs fed the PCB-only diet significantly reduced the THC and the activities of PO and ACP in the serum compared with the control (*p* < 0.05, Figure 9A,B,D). Dietary 240000 IU/kg vitamin A supplementation significantly increased the THC and PO activity in the serum compared with the crabs in the PCB-only group (*p* < 0.05, Figure 9A,B). No significant difference was observed in the activity of LZM between PCB-only group and control group. Moreover, crab in the PCB-only group had significantly lower post-challenge survival than those in the control (*P* < 0.05), and the dietary supplementation of 240000 IU/kg vitamin A increased the post-challenge survival of crabs compared with those in the PCB-only group (*p* < 0.05, Figure 9E).

## Discussion

The present study was conducted to investigate the effect of dietary PCB (Aroclor1254)-induced toxicity on growth, antioxidant capacity, immunity and energy metabolism, and alleviative the toxic effects by adding vitamin A in the diet through nutrition regulation. The results from the present study showed that dietary PCB resulted in weight loss of *E. sinensis*. Dietary supplementation of vitamin A did not significantly increase the weight gain of the Chinese mitten crab compared with that in the PCB-only group in this study. In contrast, dietary vitamin A supplementation could alleviate the weight loss induced by persistent organic pollutants in Atlantic salmon, as vitamin A could improve the impaired function of feed utilization due to the loss of intestinal enzyme function, and high energy expenditure for detoxification (Berntssen et al., 2016). But dietary supplementation vitamin A alleviated oxidative stress, immune damage, hepatopancreas lesion and energy metabolic in *E. sinensis* in present study.

Malondialdehyde is an important indicator for assessing lipid peroxidation (Zhang et al., 2015). In the present study, dietary PCB exposure significantly increased the MDA content in the serum and hepatopancreas. Similarly PCB exposure caused oxidative damage in chicken embryos and rats (Katynski et al., 2004; Shen et al., 2014). Present research has shown that vitamin A reduced lipid peroxidation caused by Aroclor 1254, for vitamin A is an effective antioxidant in reducing oxidative stress (Zhou and Zhang, 2005). In the present study, dietary 80000 IU/kg vitamin A supplementation also alleviated the PCB-induced lipid peroxidation in both serum and hepatopancreas. In the present study, dietary PCB exposure increased the SOD activity in serum and hepatopancreas, and the SOD activity was reduced in crab fed vitamin A supplementation. Similarly, superoxide dismutase can convert superoxide into hydrogen peroxide and consequently protect organisms from oxidative damage (Wang et al., 2014; Lin et al., 2018). The possible mechanism is that vitamin A, as a non-enzymatic antioxidant, can scavenge free radicals in the body, and vitamin A is involved in the detoxification of PCB in the hepatopancreas (Berntssen et al., 2016). The CAT activity in the serum increased in crab fed the PCB-only diet compared with the control group, and dietary vitamin A addition effectively improved this condition. The induction of CAT activity is related to oxidative stress leading to the continuous generation of H_2_O_2_ from ${\text{O}}_{2}^{-}$, and the activated CAT activity could protect the organism by scavenging H_2_O_2_ (Wu et al., 2018). In the hepatopancreas, the GPx activity was significantly decreased by dietary PCB exposure, but dietary vitamin A supplementation could not relieve the negative effects. It is speculated that glutathione, which is required for GPx synthesis, was not only involved in the synthesis of GPx, but also involved in GST synthesis and PCB metabolism (Venkataraman et al., 2007; Song et al., 2009).

In the present study, dietary PCB exposure increased the activity of phase I detoxification enzyme ERND by inducing the up-regulation of the CYP gene mRNA expression of hepatopancreas. In addition, the activity of phase II detoxification enzyme GST also significantly increased, indicating metabolic enhancement of PCB in *E. sinensis*. For PCB can be metabolized in the P450 detoxification enzyme system (Snyder, 2000). Moreover, PCB and vitamin A could be metabolized in the same metabolic pathway, therefore the addition of vitamin A in the diet could affect the metabolism of PCB (Yang et al., 2005). In the present study, dietary vitamin A supplementation had no significant effect on the expression of the CYP2 in the hepatopancreas, but the supplementation of 240000 IU/kg vitamin A significantly reduced the CYP4 mRNA level in the hepatopancreas compared with those in the PCB-only group. It is possible that the CYP2 gene was not only involved in the metabolism of PCB, but also in the metabolism of vitamin A, leading to a relatively high level of CYP2 mRNA. Meanwhile, CYP4 may be also closely related to PCB metabolism. Findings from other studies showed that dietary vitamin A supplementation can decrease the up-regulation of CYP genes induced by PCB in Atlantic salmon (Arukwe and Nordbø, 2008).

Hepatopancreas is an important organ for detoxification. The PCB-only group showed that many B cells appeared in the hepatopancreas tubules, which could transfer metabolic wastes out of the cells under stress. In contrast, the hepatopancreas cells of the control group were closely arranged and R cells was dominant. It indicates that no obvious damage in the hepatopancreas of *E. sinensis*. Furthermore, the results showed that dietary 80000 IU/kg vitamin A supplementation could effectively protect hepatopancreas from PCB damage, which may be explained by the use of appropriate amount of vitamin A as an antioxidant in the diet to reduce the level of oxidative stress in *E. sinensis*. Similarly, antioxidant compounds increased LB400 cells integrity after PCB exposure, for preventing direct oxidative damage mediated by ROS (Ponce et al., 2011). However, there were some lesions on hepatopancreas in the PVA240K group compared with that in the PVA80K group. The reason could be that excessive vitamin A may affect the clearance of free radicals, or vitamin A may accelerate the metabolism of PCB, producing a large amount of terpenoids, and thereby leading to massive production of ROS. This also explains why the MDA content was increased in the hepatopancreas in the PVA240K group compared with the crabs in the PVA80K group.

The present results of oil-red O staining showed that dietary vitamin A supplementation increased lipid deposition what decreased by dietary PCB exposure. Because vitamin A is an antioxidant, it can prevent the body from lipid peroxidation, and reduce the energy required to cope with PCB stress. The expression of FABP3, FABP10, and IL genes in the hepatopancreas were down-regulated when vitamin A was supplemented in the diet, suggesting that the lipid hydrolysis for energy supply is reduced, leading to an increase of lipid deposition in Chinese mitten crab. Similar results were found in another study, vitamin A as an important regulator of lipid metabolism reduce the level of transcriptional activator STAT mRNA through the retinoic acid pathway and inhibit insulin signaling pathway and PPAR pathway to increase lipid accumulation (Berry et al., 2011). The present study showed that both dietary PCB and vitamin A supplementation significantly increased the mRNA expression of the RXR gene in the hepatopancreas. It may be explained by the fact that up-regulation of the RXR gene can activate PPAR/RXR-mediated lipid metabolism and accelerate PCB metabolism (Hardwick et al., 2009).

In the present study, dietary PCB exposure also decreased the THC level, suggesting that the immunity of *E. sinensis* may be decreased by dietary PCB exposure. The THC and activities of PO and ACP have been used to evaluate the health condition of aquatic animals (Dong et al., 2009; Wei et al., 2014). The haemocyte in crustaceans is a crucial part of the cellular immune system for melanization, cytotoxicity, cell recognition and phagocytosis (Johansson et al., 2000). The environmental stress from sulfide, copper, salinity, nitrite or ammonia can reduce THC in crustaceans (Liu et al., 2004; Hong et al., 2007; Joseph and Philip, 2007), and low THC may impair immune capability and decrease the ability of disease or stress resistance (Xian et al., 2011). Meanwhile, due to the lack of an adaptive immune system, PO play a key role for defending pathogens and oxidative stress in a crustacean (Dong et al., 2009). ACP is the typical hydrolases involved in the extermination of toxin invasion and pollutant detoxification, and also plays a positive role in the immune system of crustaceans as part of the lysosomal enzyme (Zhao et al., 2014). The present study revealed that dietary PCB exposure reduced the serum PO and ACP activities, indicating a decline in the resistance of Chinese mitten crab to cope with pathogens (Mottin et al., 2010). Moreover, dietary vitamin A could alleviate the reduction of THC and PO activity induced by dietary PCB exposure, indicating that dietary vitamin A improve the immunity of *E. sinensis* exposed to PCB. In addition, the changes of THC, serum PO and ACP activities could result in the change in disease resistance of *E. sinensis*. The present study suggests that the disease resistance of *E. sinensis* will decrease when the crabs are exposed to PCB, but dietary 240000 IU/kg vitamin A supplementation can improve disease resistance of *E. sinensis*.

## Conclusion

In conclusion, vitamin A plays important physiological functions in crustacean. Findings from the present study have shown that dietary 80000 IU/kg vitamin A supplementation could provide protection against the adverse impacts of PCB exposure, while 240000 IU/kg vitamin A supplementation could effectively enhance the immunity of *E. sinensis*.

## Data Availability

All datasets for this study are included in the manuscript and the supplementary files.

## Ethics Statement

The use of animals in this study was approved by the Committee on the Ethics of Animal Experiments of East China Normal University.

## Author Contributions

DF and LC designed the experiment. DF and XW analyzed the experimental results and wrote the manuscript. EL, XB, JQ, and FQ helped in correcting the manuscript. All authors have given approval to the final version of the revised manuscript.

## Conflict of Interest Statement

The authors declare that the research was conducted in the absence of any commercial or financial relationships that could be construed as a potential conflict of interest.
